# Supplementary material for: Successful Dendrimer and Liposome-Based Strategies to Solubilize an Antiproliferative Pyrazole Otherwise Not Clinically Applicable
Source: Nanomaterials (Basel). 2022 Jan 11;12(2):233. doi: 10.3390/nano12020233 (PMC8780786; doi:10.3390/nano12020233)
Supplement: Supplementary file 1 [file nanomaterials-12-00233-s001.zip › nanomaterials-1533391-Supplementary.pdf]

nanomaterials

Supplementary Materials

Successful Dendrimer and Liposome-Based Strategies to Solubilize an Antiproliferative Pyrazole Otherwise Not Clinically Applicable

Silvana Alfei *, Andrea Spallarossa, Matteo Lusardi and Guendalina Zuccari

Department of Pharmacy, University of Genoa, Viale Cembrano, 16148 Genoa, Italy; andrea.spallarossa@unige.it (A.S.); matteo.lusardi@edu.unige.it (M.L.); zuccari@difar.unige.it (G.Z.)
* Correspondence: alfei@difar.unige.it; Tel.: +39-010-355-2296

Section S1

S1.1. ATR-FTIR data of 3-(4-chlorophenyl)-5-[(4-nitrophenyl)amino]-1H-pyrazole-4-carbonitrile (CR232)

Orange crystals (63% isolated yield), m.p. > 300 °C (diethyl ether/petrol ether). FTIR (KBr, ν, cm^{-1}): 3324 (NH), 3223, 3167, 3136 (H-C= stratching aromatic rings), 2224 (CN), 1600 (CH=CH stretching phenyl rings), 1486, 1327 (NO_2 group).

S1.2. Copies of ATR-FTIR, ^1H NMR and ^{13}C NMR spectra of CR232

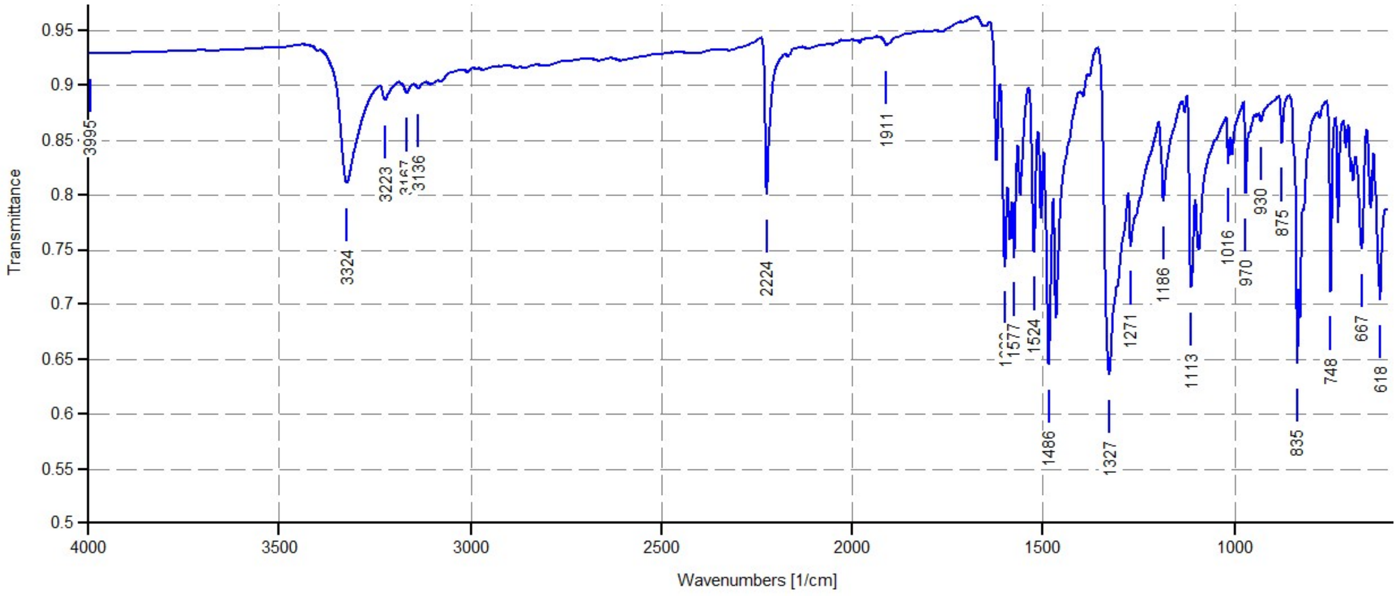

Figure S1. ATR-FTIR spectrum of CR232.

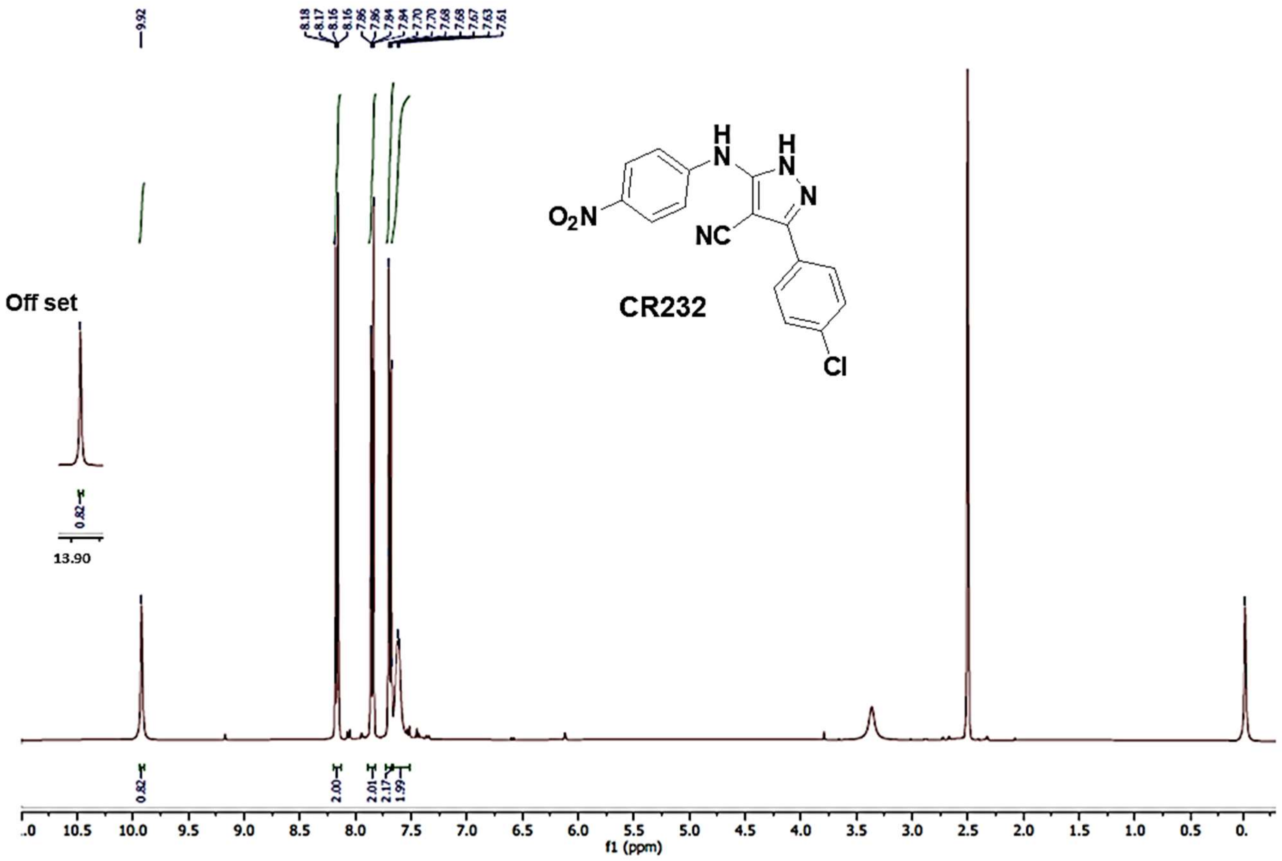

Figure S2. ^{1}H NMR (DMSO-*d6*, 400 MHz) of CR232.

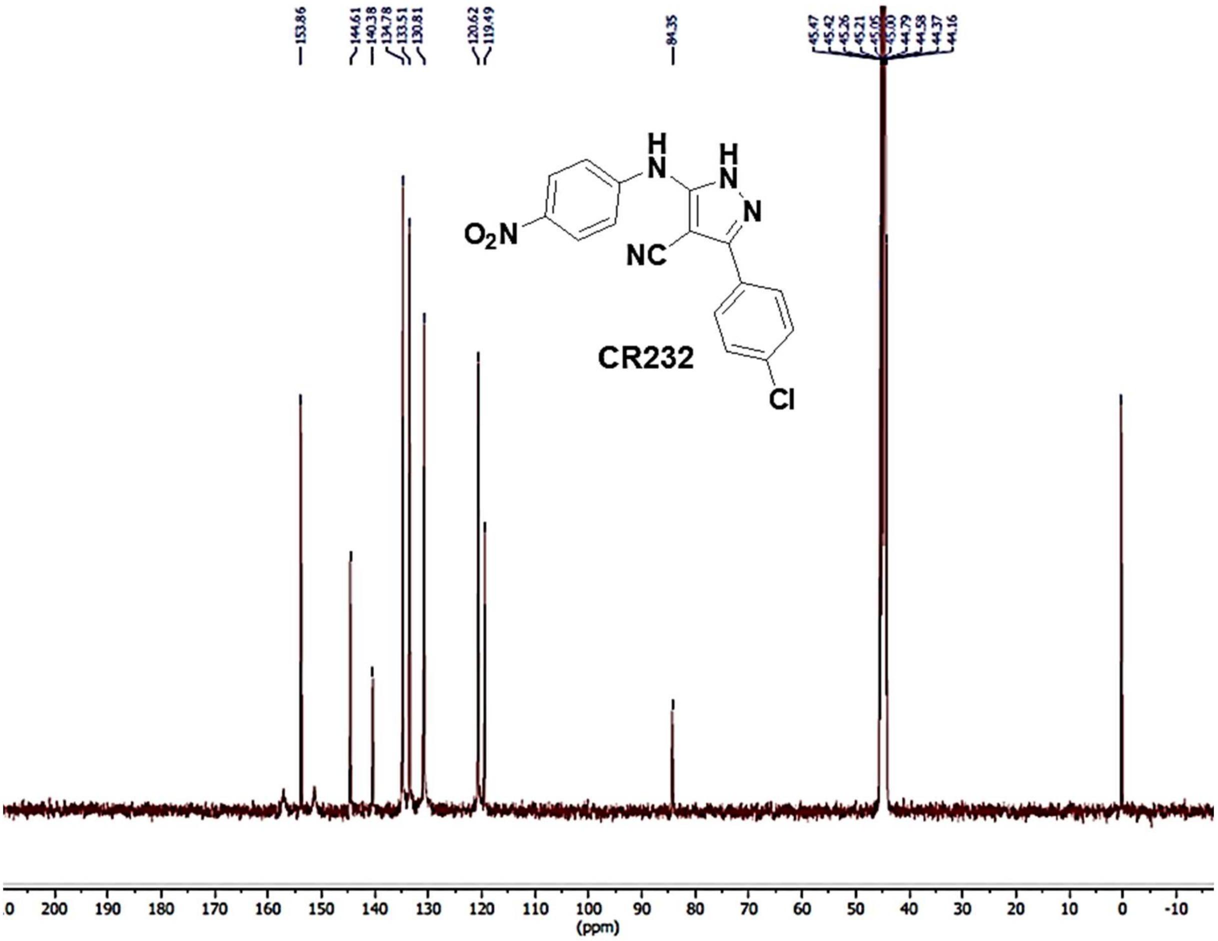

Figure S3. [13]H NMR (DMSO-*d6*, 100 MHz) of CR232.

Section S2

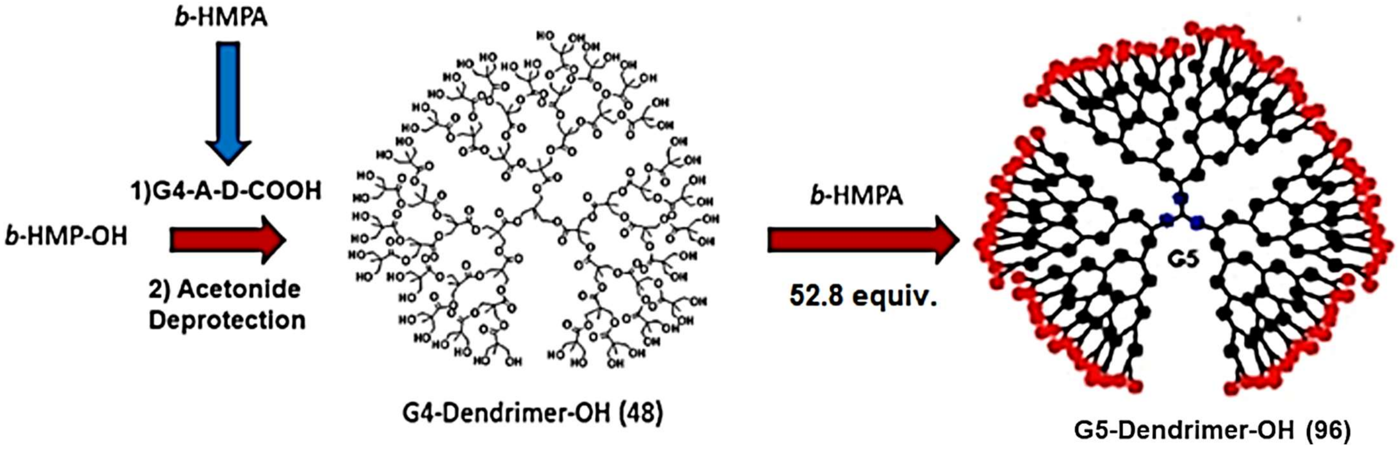

Scheme S1. Synthetic route to prepare the uncharged dendrimers G4OH and G5OH. D = dendron (a single chemically addressable group called the focal point or core); G4 and G5 indicate the number of generations; 48 and 96 are the number of peripheral hydroxyl groups; red spheres = 96 OH groups.

S2.1. G4OH

Fluffy white solid (98% isolated yield), m.p. 77 °C. FTIR (KBr, ν, cm^{-1}): 3424 (OH), 1739 (C=O). ^{1}H NMR (DMSO-*d6*, 400 MHz): δ = 0.80 (s, 3H, CH_3 of core), 1.01 (s, 72H, CH_3 of fourth generation (G4)), 1.16 (s, 36H, CH_3 of third generation (G3)), 1.18 (s, 18H, CH_3 of second generation (G2)), 1.22 (s, 9H, CH_3 of first generation (G1)), 3.29-3.49 (m, 96H, CH_2OH); 4.08-4.30 (m, 90H, CH_2O of dendrimer), 4.55 (br q, 48H, OH). ^{13}C NMR (DMSO-*d6*, 100 MHz): δ = 16.67, 16.84, 16.88, and 17.12 (CH_3), 46.16, 46.19, 46.23 and 50.20 (quaternary C), 63.63 (CH_2OH), 64.33, 64.86 and 65.29 (CH_2O), 171.42 (two signals overlapped), 171.79 and 174.00 (C=O), CH_3, quaternary C and CH_2O of core were no detectable. Anal. Cald. for $C_{230}H_{372}O_{138}$ requires C, 51.68; H, 7.01%. Found: C, 51.86; H 7.18.

S2.2. G5OH

White fluffy solid (99% isolated yield). FTIR (KBr, ν, cm^{-1}): 3421 (OH), 1736 (C=O). ^{1}H NMR (DMSO-*d6*, 400 MHz): δ = 0.88 (s, 3H, CH_3 of core), 1.00, 1.01, 1.06, 1.16, 1.23 (five s, 279H, CH_3 of dendrimer generations), 3.41-3.44 (m, 192 H, CH_2OH), 4.00-4.20 (m, 186H, CH_2O of dendrimer), 4.60-5.00 (br, 96H, OH). ^{13}C NMR (DMSO-*d6*, 100 MHz): δ = 16.67-16.93 (CH_3 of dendrimer), 46.05-50.10 (quaternary C of dendrimer), 63.55 (CH_2O of dendrimer), 171.52-176.55 (C=O), CH_3, quaternary C and CH_2O of core were no detectable. Anal. Cald. for $C_{470}H_{756}O_{282}$ requires C, 51.70; H, 6.98%. Found: C, 51.66; H, 7.09.

Section S3

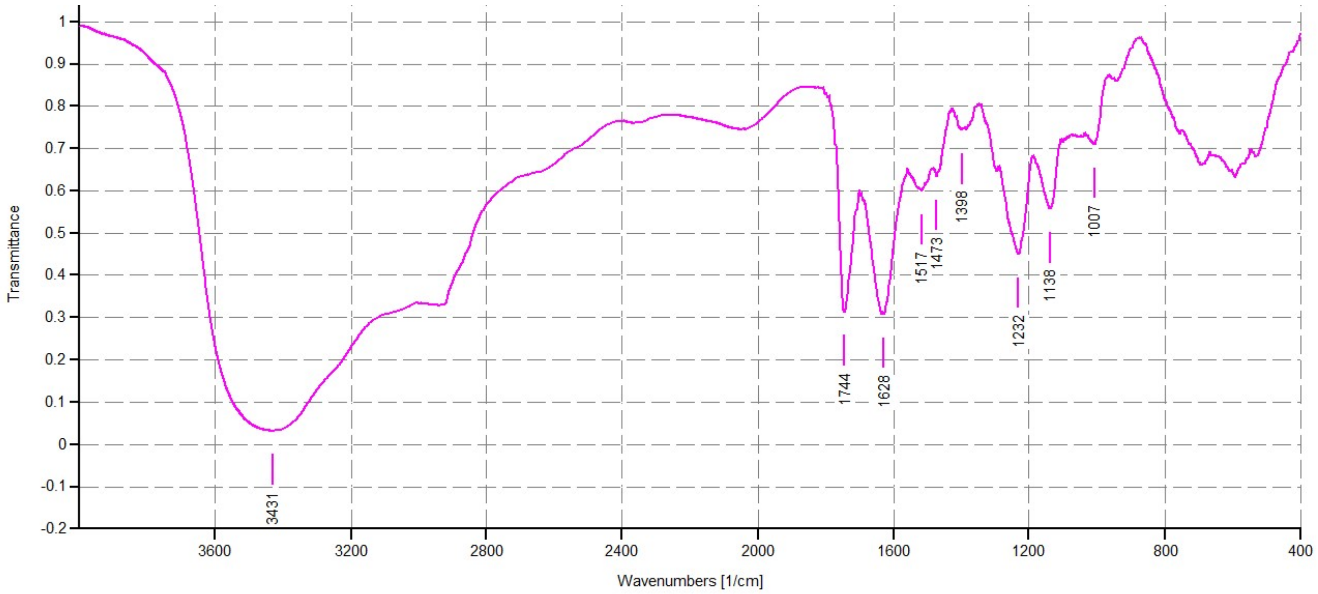

Figure S4. ATR-FTIR spectrum of G5K.

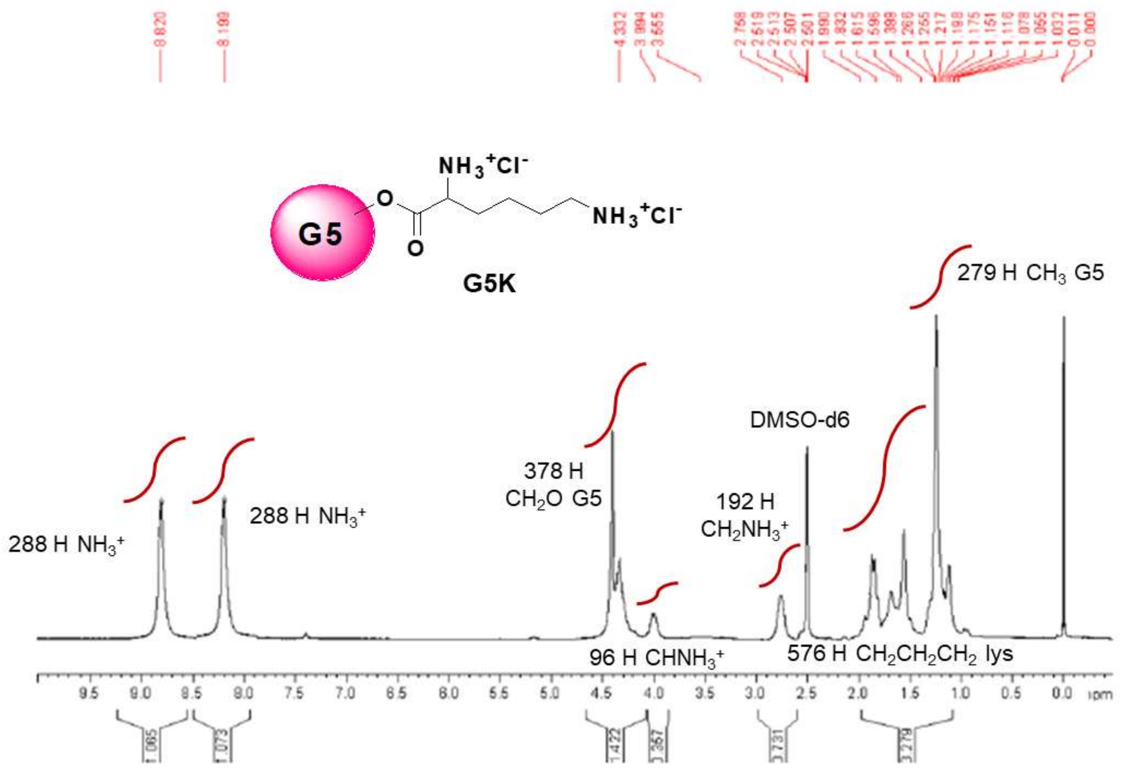

Figure S5. ^{1}H NMR (DMSO-*d6*, 400 MHz) of G5K.

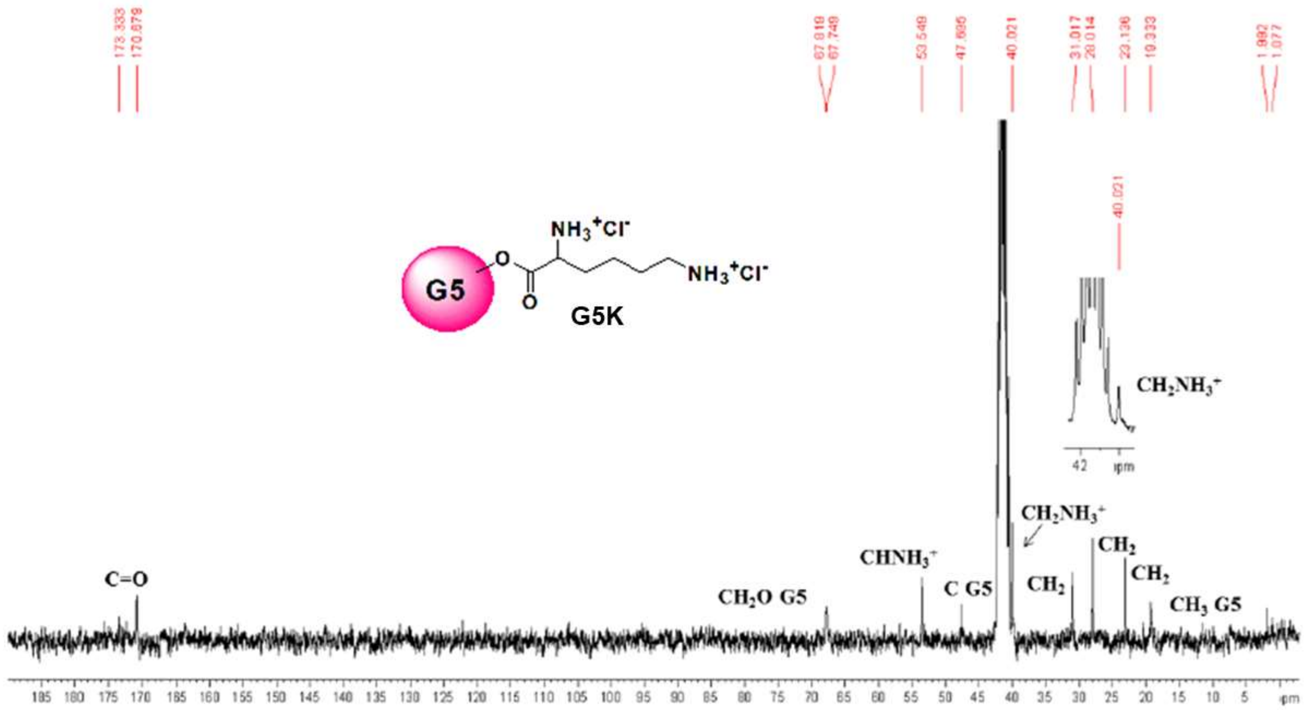

Figure S6. ^{13}C NMR (DMSO-*d6*, 100 MHz) of G5K.

Section S4

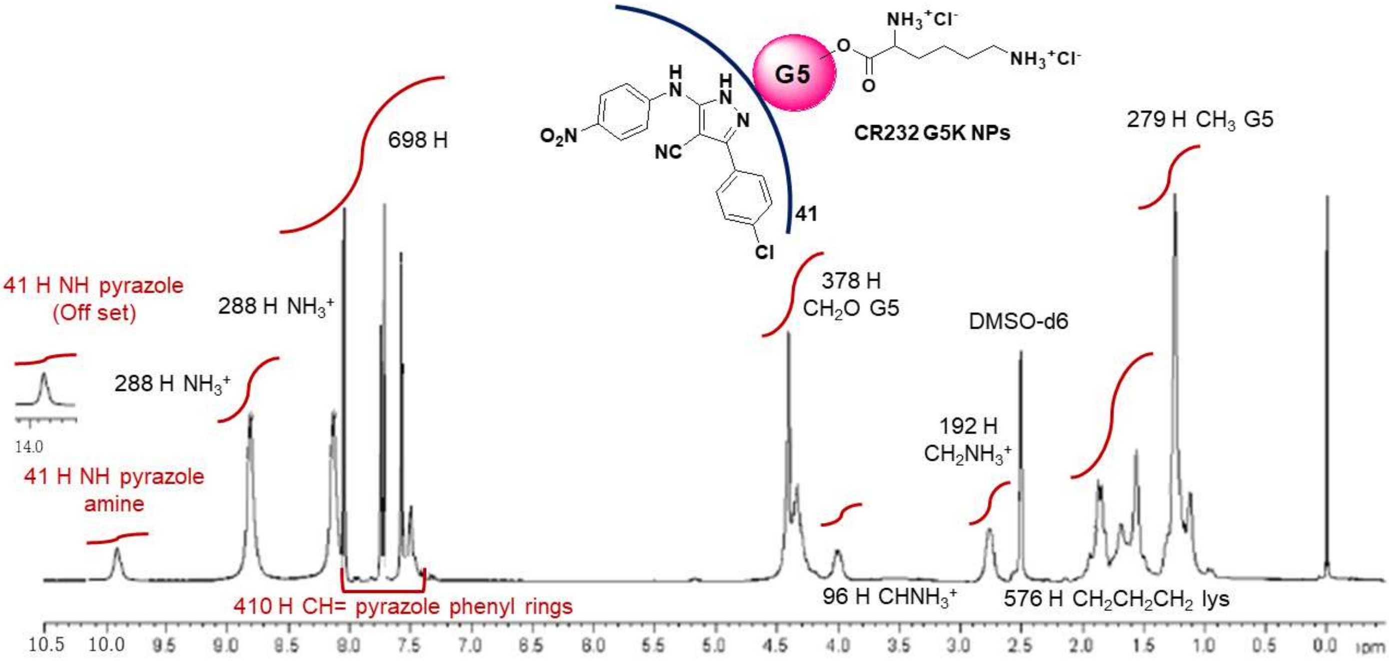

Figure S7. ^{1}H NMR spectrum (DMSO-*d6*, 400 MHz) of CR232-G5K NPs.

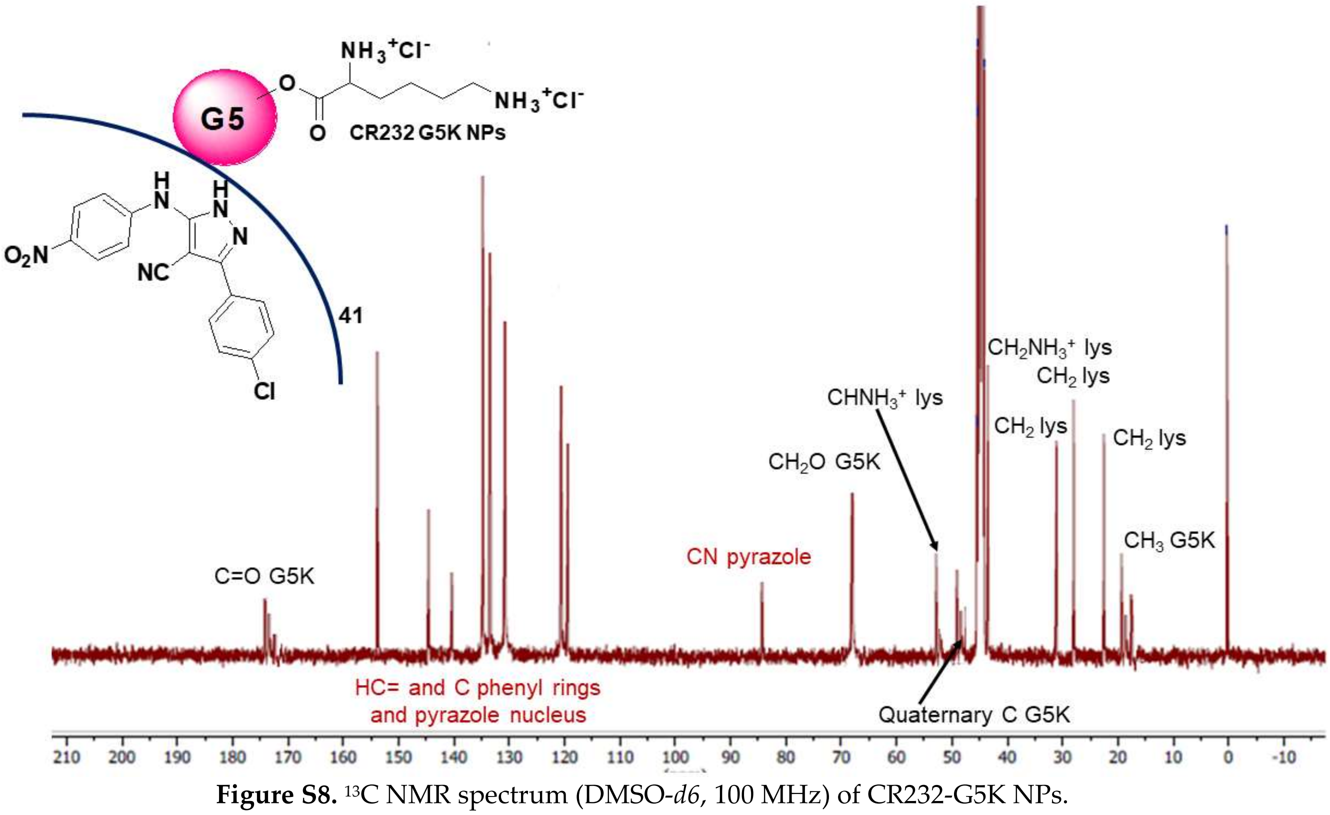

Figure S8. ^{13}C NMR spectrum (DMSO-*d6*, 100 MHz) of CR232-G5K NPs.

Section S5

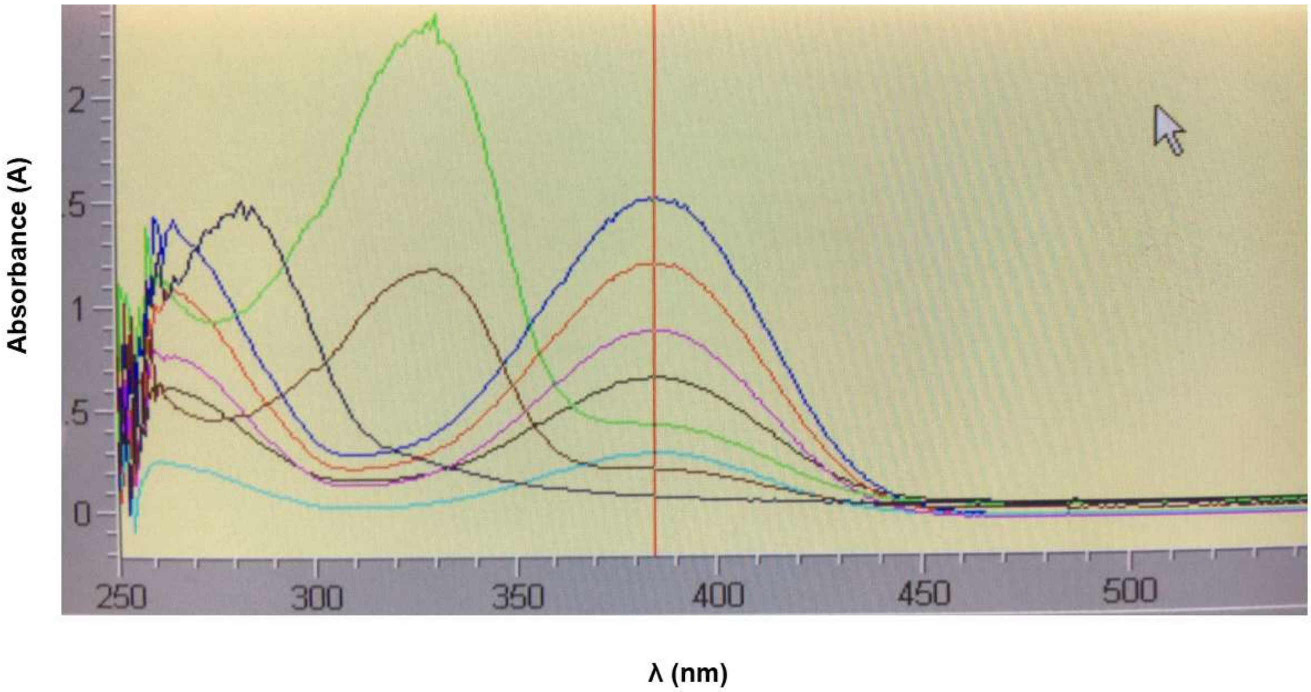

Figure S9. UV-Vis spectra of G5K (black line around λ_{abs} = 280 nm), of CR232-G5K complex (brown and light green lines at λ_{abs} = 328 nm), and of CR232 (all other lines with λ_{abs} = 254 and 384 nm).

Section S6

Table S1. Data of the calibration curve: [A], C_{CR232}, C_{CR232p}, residuals, and absolute errors (%).

C_{CR232} (mg/mL)	[A] (mAU)	C_{CR232p} (mg/mL)	Residuals (mg/mL)	Absolute errors (%)
0.02414	1.5156±0.0346	0.02507	+0.00093	4.2
0.01995	1.1997±0.0421	0.01918	-0.00014	3.9
0.01496	0.8827±0.0231	0.01453	-0.00043	2.9
0.00998	0.6552±0.0421	0.01073	+0.00075	7.6
0.00499	0.2909±0.0501	0.00468	-0.00030	6.2

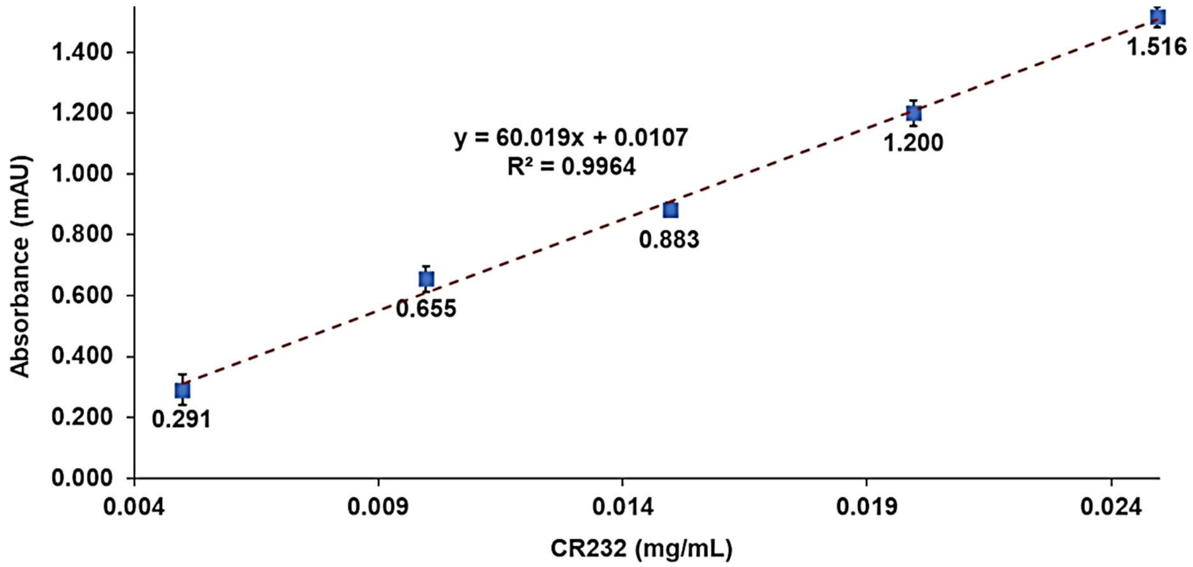

Figure S10. CR232 linear calibration model.

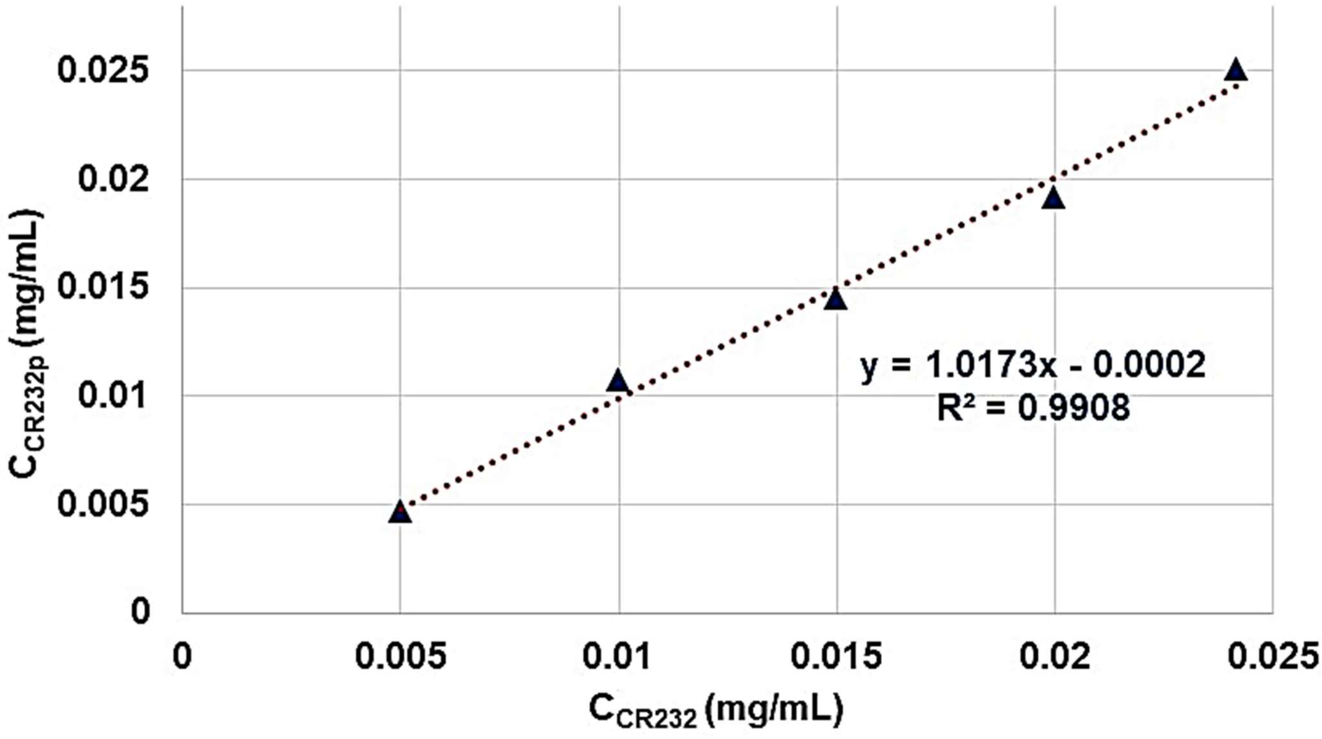

Figure S11. Linear regression of CR232 concentrations predicted by the calibration model (C_{CR232p}) vs. standard concentrations of CR232 (C_{CR232}).

Section S7

Table S2. Values of the coefficients of determinations R^2 of the linear regressions associated to the dispersion graphs obtained fitting the different mathematical models to the CR% curve data.

Mathematical Model	R^2	R^2
	CR232-G5K NPs	CR232-SUVs 30/1
Zero Order	0.8682	0.9601
First Order	0.9487	0.9569
Hixson-Crowell	0.6468	N.T.
Higuchi	0.8990	0.9353
Korsmeyer-Peppas	0.9458	0.9454
Weibull	0.9754	0.9407

N.T. = not tested, because rarely considered in literature for liposomes-based formulations.

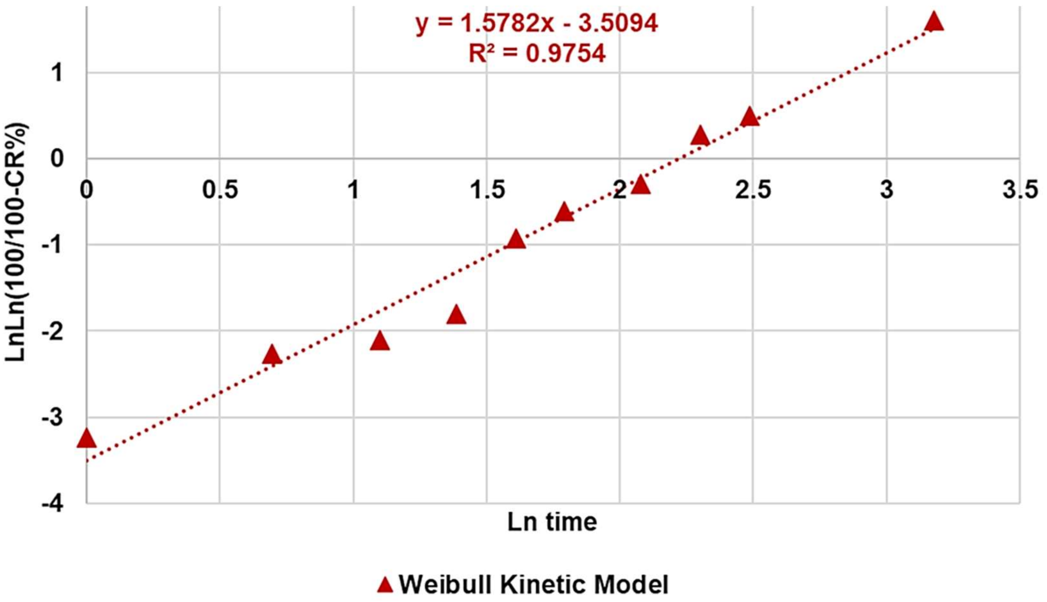

Figure S12. Linear regression of Weibull kinetic mathematical model with the related equation and R^2 value.

Section S8

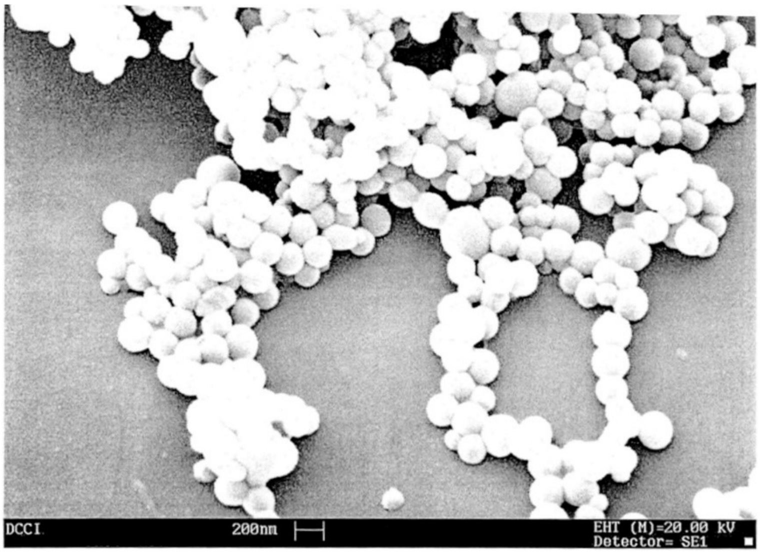

(**a**)

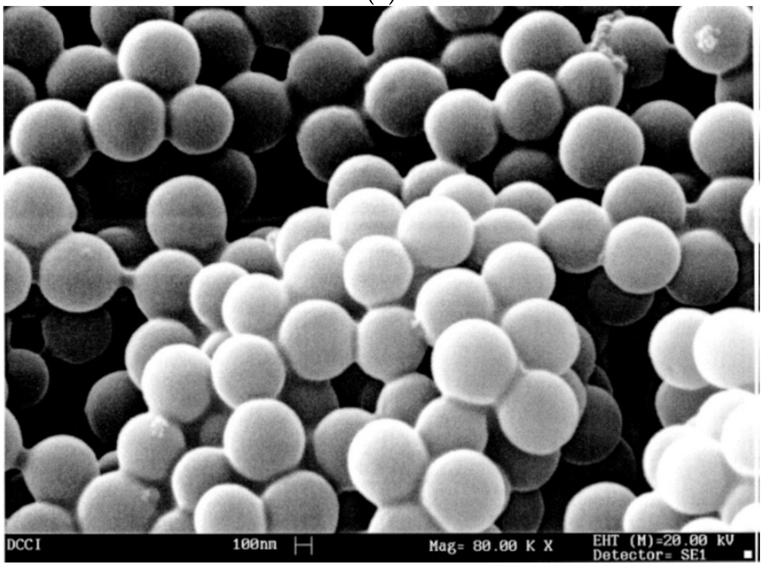

(**b**)

Figure S13. SEM images of G5K (**a**) and CR232-G5K (**b**) particles.

Section S9

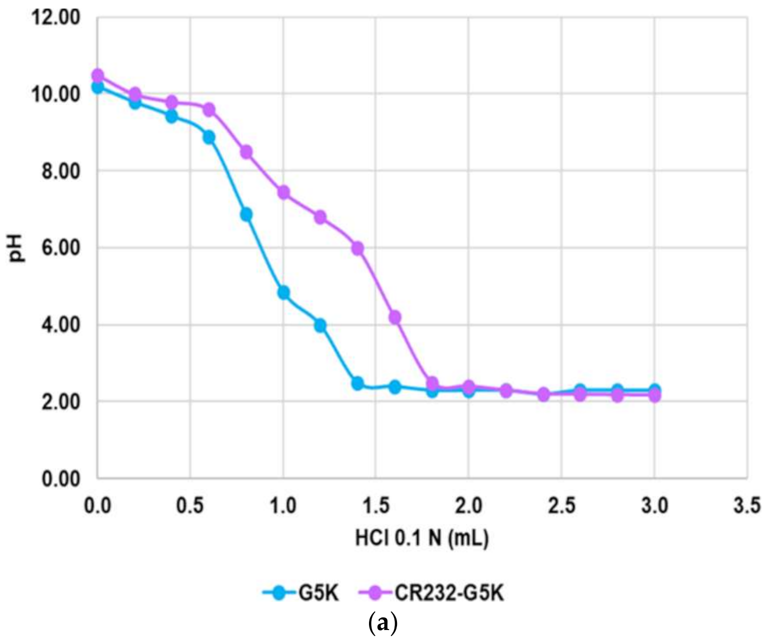

(**a**)

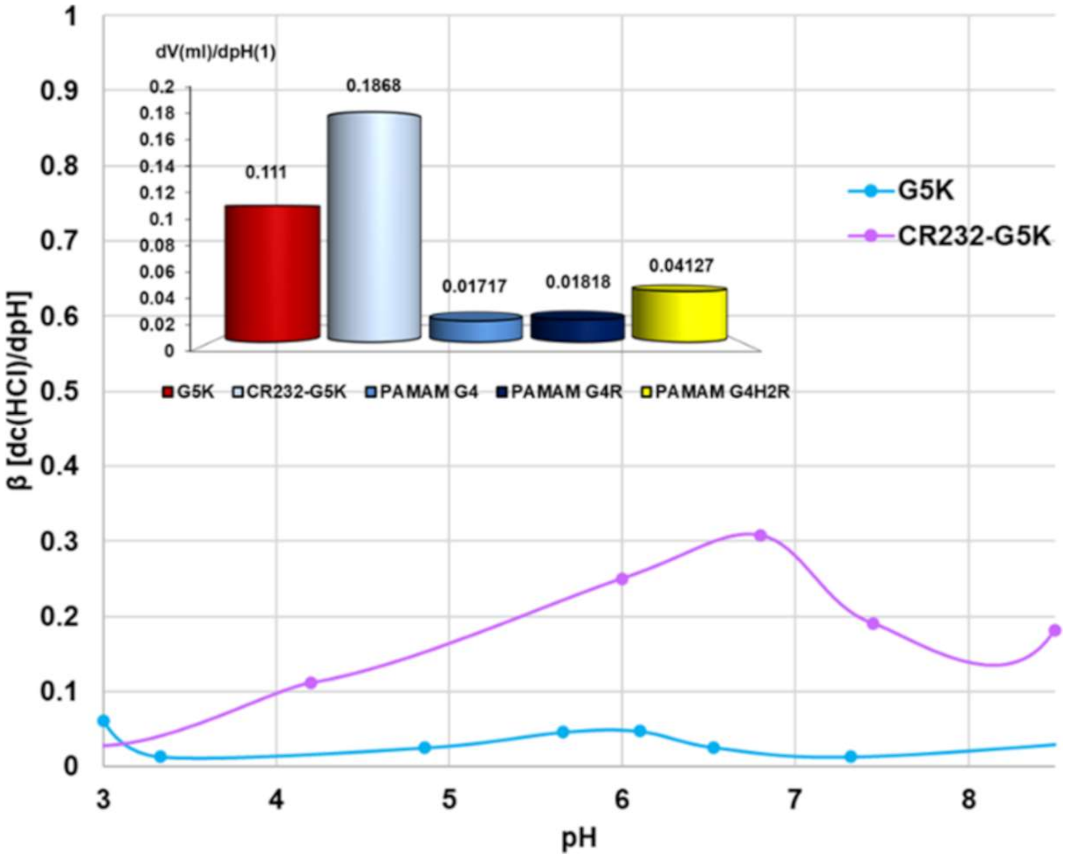

(**b**)

Figure S14. Titration curves (error bars not reported since difficult to detect (**a**), β values vs. pH values and values of β mean presented as bars graph of CR232-G5K NPs and of three PAMAM of fourth generation for comparison (**b**).

Section S10

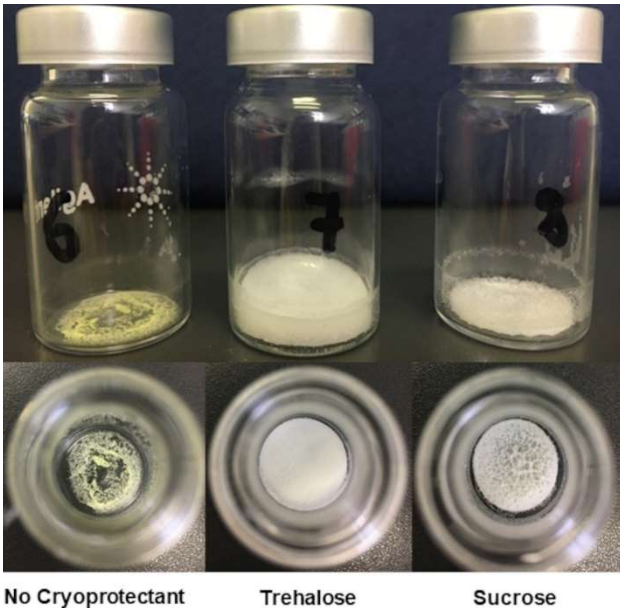

Figure S15. Solid liposomes obtained by freeze-drying the CR232-SUV suspension 30/1, without cryoprotectant (glass container 6), with trehalose (glass container 7), and with sucrose (glass container 8).

Section S11

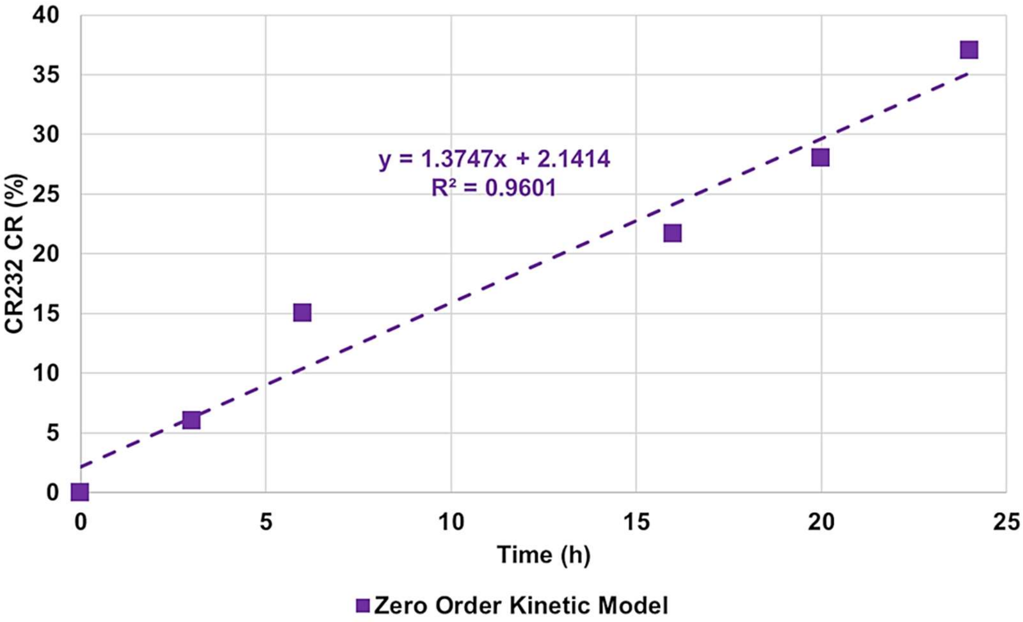

Figure S16. Linear regression of zero order kinetic mathematical model with the related equation and R^2 value.

Section S12

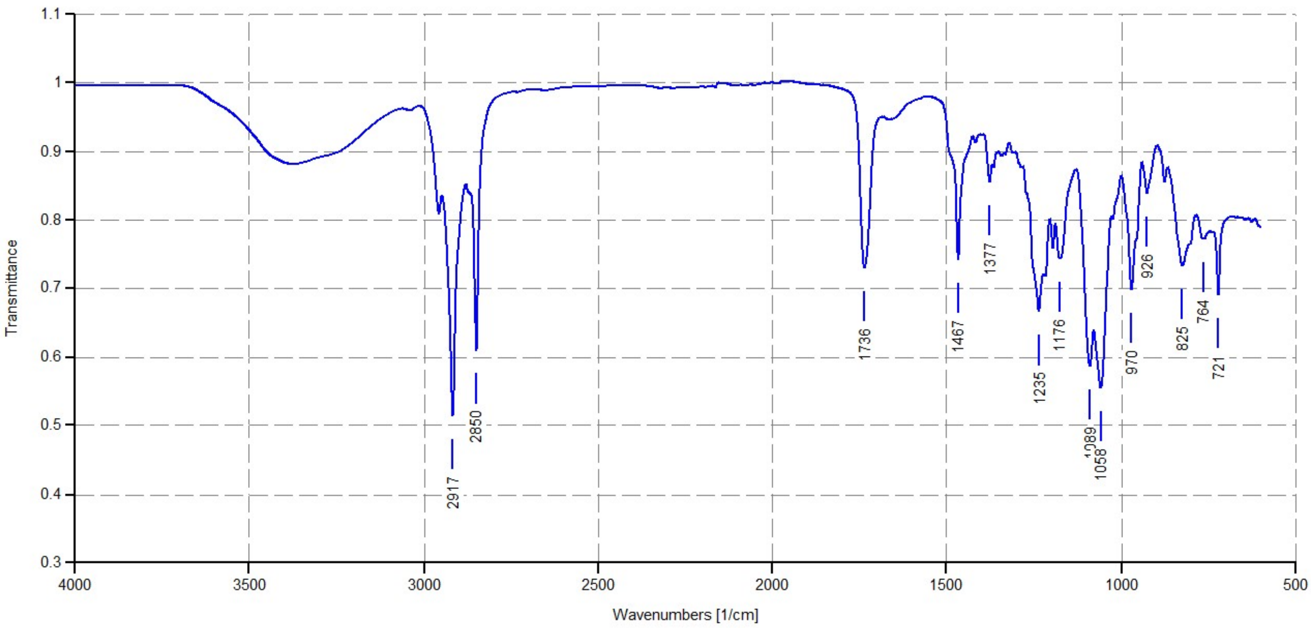

Figure S17. ATR-FTIR of SUVs (empty liposomes).

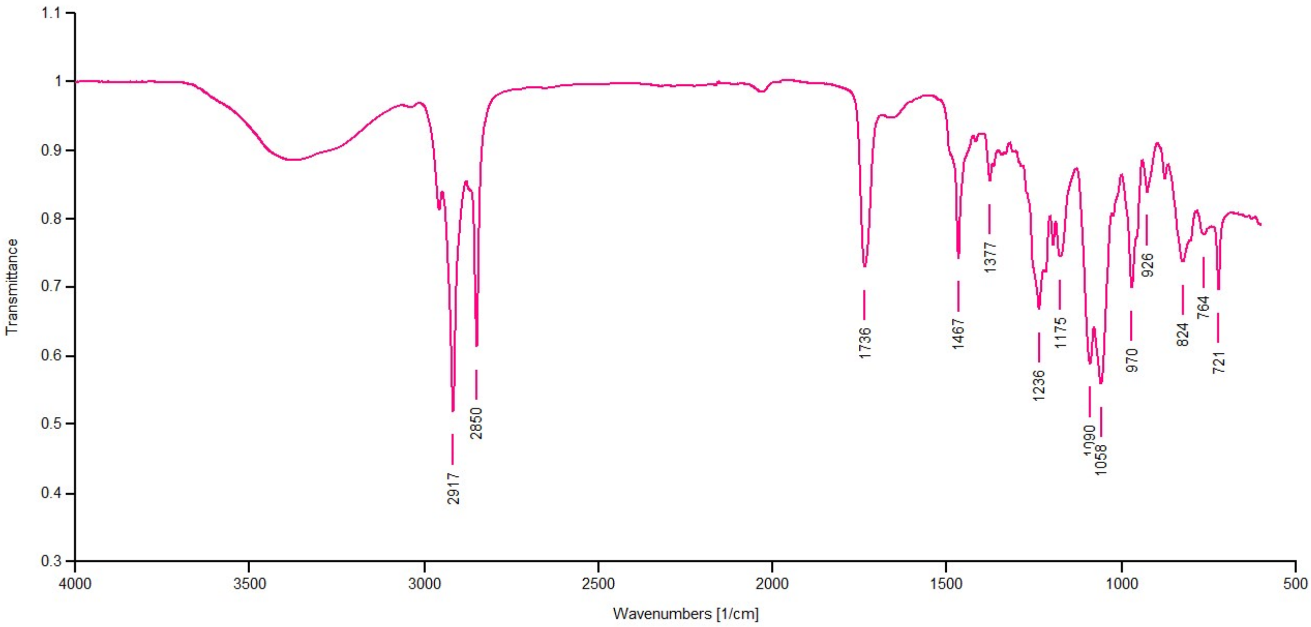

Figure S18. ATR-FTIR of CR232-SUVs 5/1 (ratio lipids/CR232). Since lipids concentration was maintained constant, 5/1 formulation was the CR232-liposomes formulation prepared with the highest initial amount of CR232, which proved the lowest EE%. The band at 2225 cm^{-1} (typical of CN group of CR232) is not detectable.

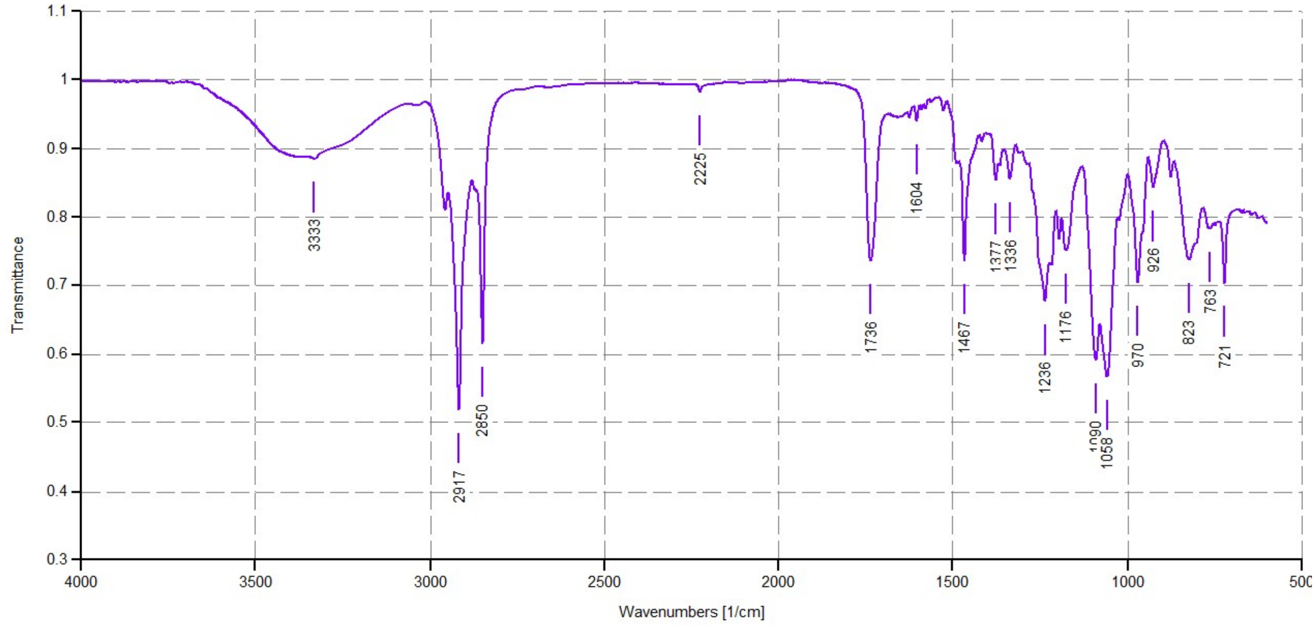

Figure S19. ATR-FTIR of CR232-SUVs 15/1 (ratio lipids/CR232). Since lipids concentration was maintained constant, 15/1 formulation was the CR232-liposomes formulation prepared with the intermediate initial amount of CR232, which proved the intermediate EE%. A small band at 2225 cm^{-1} (typical of CN group) of CR232 is detectable.

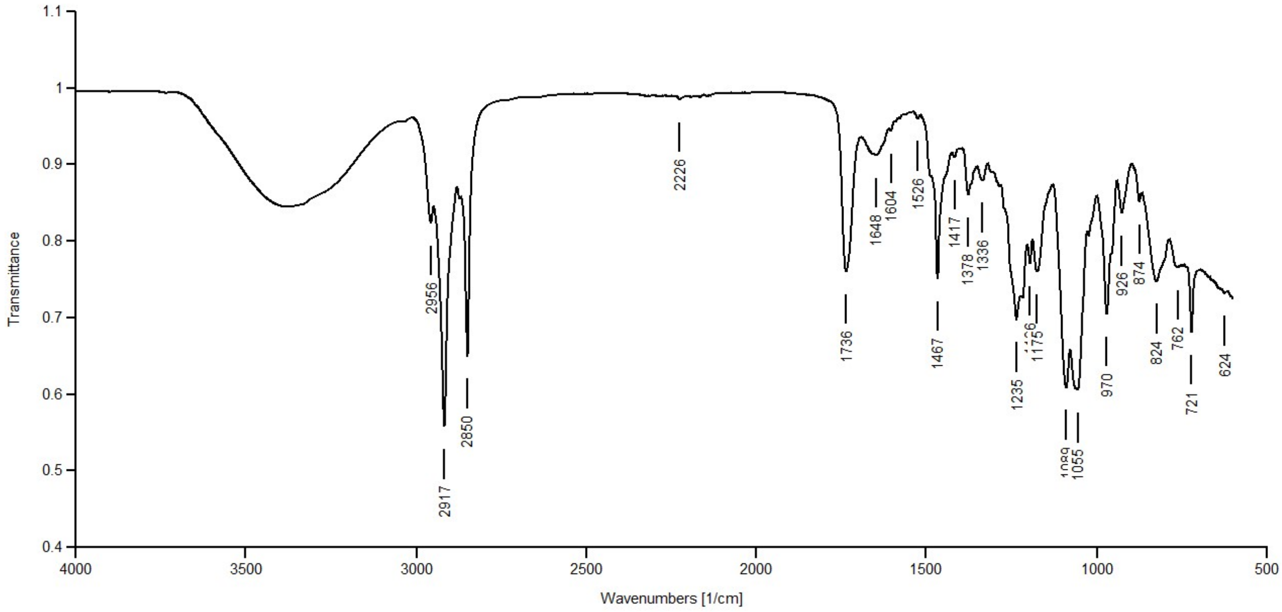

Figure S20. ATR-FTIR of CR232-SUVs 30/1 (ratio lipids/CR232). Since lipids concentration was maintained constant 30/1 formulation was the CR232-liposomes formulation prepared with the lowest amount of CR232, which proved the highest EE%. A very small band at 2226 cm^{-1} (typical of CN group of CR232) is detectable.

Section S13

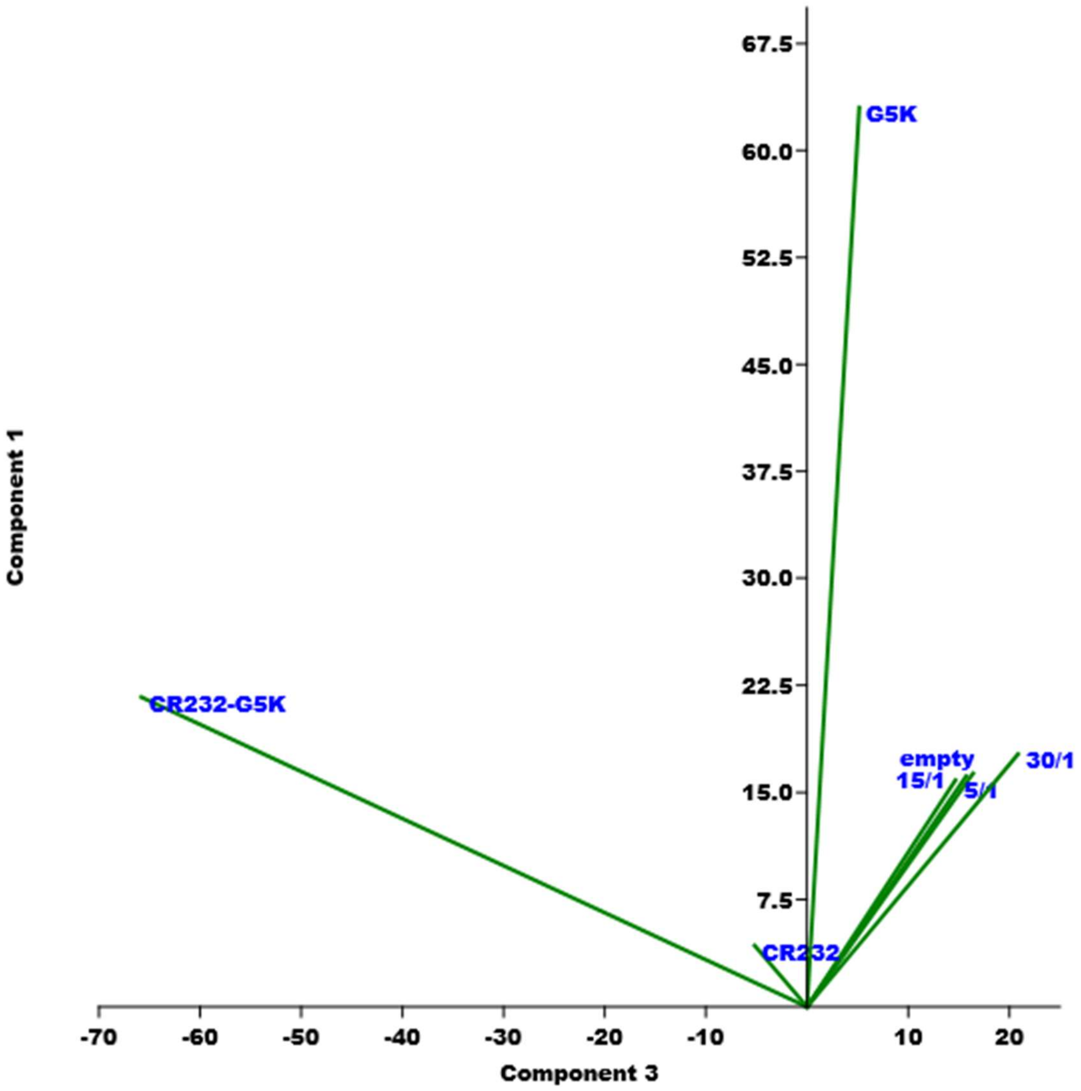

Figure S21. Principle component analysis (PCA) results (represented as a score plot) performed on the matrix collecting spectral data of CR232, G5K, SUVs, CR232-G5K and CR232-SUVs (PC1 vs. PC3).

Section S14

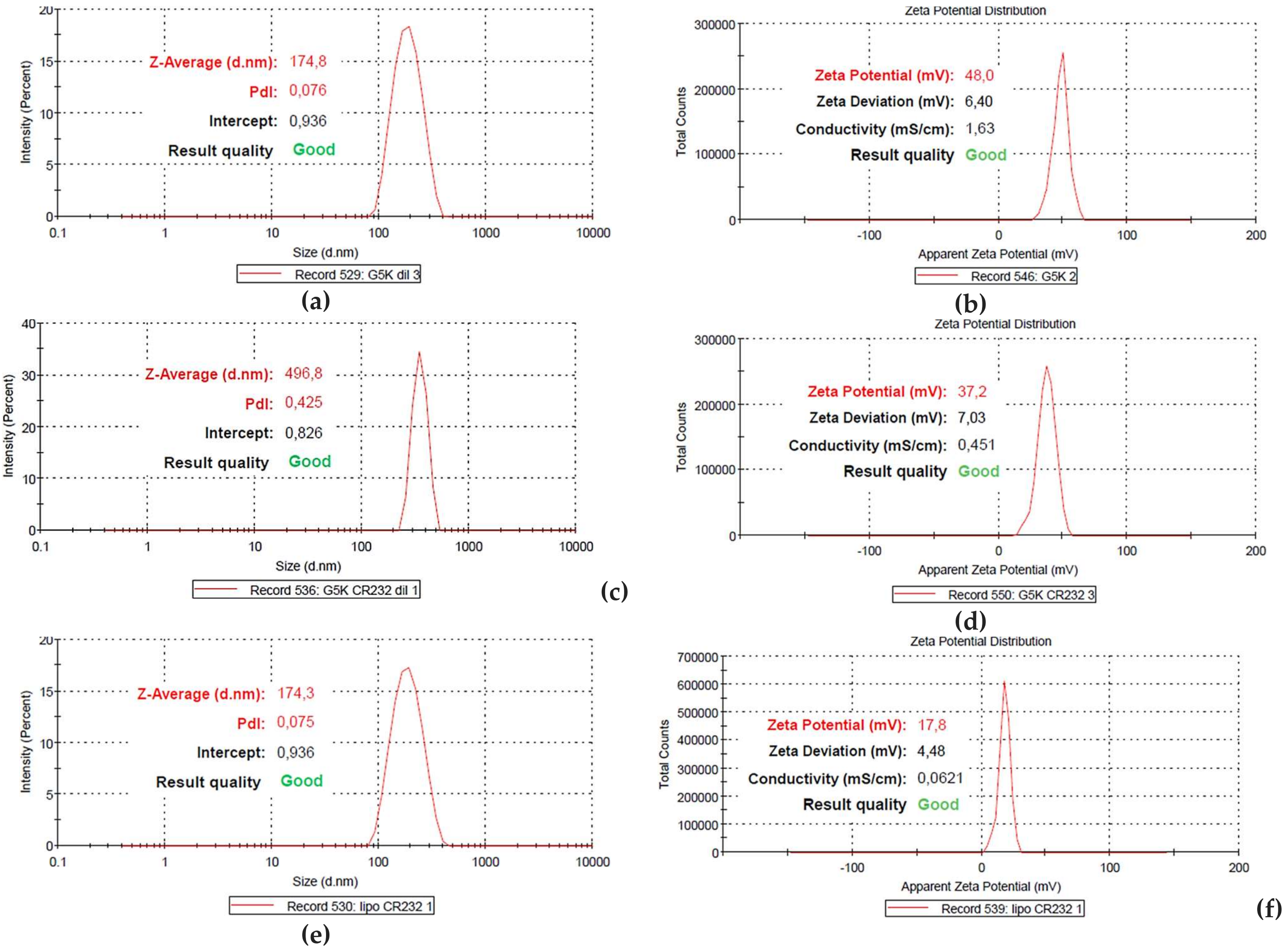

Figure S22. Representative particle size distributions of G5K (**a**) CR232-G5K (**c**) and of CR232-SUVs (**e**), and representatives ζ-p distributions of G5K (**b**) CR232-G5K (**d**) and of CR232-SUVs (**f**).
